# A deleterious mutation in the ALMS1 gene in a naturally occurring model of hypertrophic cardiomyopathy in the Sphynx cat

**DOI:** 10.1186/s13023-021-01740-5

**Published:** 2021-02-27

**Authors:** Kathryn M. Meurs, Brian G. Williams, Dylan DeProspero, Steven G. Friedenberg, David E. Malarkey, J. Ashley Ezzell, Bruce W. Keene, Darcy B. Adin, Teresa C. DeFrancesco, Sandra Tou

**Affiliations:** 1 Department of Veterinary Clinical Sciences, North Carolina State University, Raleigh, NC 27607 USA; 2 Department of Veterinary Clinical Sciences, University of Minnesota, Saint Paul, MN 55108 USA; 3 National Toxicology Program Pathology Group, Cellular and Molecular Pathology Branch, National Institute of Environmental Health Sciences, Research Triangle Park, Durham, NC 27709 USA; 4 Histology Research Core Facility, Department of Cell Biology and Physiology, University of North Carolina, Chapel Hill, NC 27599 USA

**Keywords:** Hypertrophic cardiomyopathy, ALMS1, Feline, Mitogenic, Sphynx

## Abstract

**Background:**

Familial hypertrophic cardiomyopathy is a common inherited cardiovascular disorder in people. Many causal mutations have been identified, but about 40% of cases do not have a known causative mutation. Mutations in the *ALMS1* gene are associated with the development of Alstrom syndrome, a multisystem familial disease that can include cardiomyopathy (dilated, restrictive). Hypertrophic cardiomyopathy has not been described. The *ALMS1* gene is a large gene that encodes for a ubiquitously expressed protein. The function of the protein is not well understood although it is believed to be associated with energy metabolism and homeostasis, cell differentiation and cell cycle control. The ALMS1 protein has also been shown to be involved in the regulation of cell cycle proliferation in perinatal cardiomyocytes. Although cardiomyocyte cell division and replication in mammals generally declines soon after birth, inhibition of ALMS1 expression in mice lead to increased cardiomyocyte proliferation, and deficiency of Alstrom protein has been suggested to impair post-natal cardiomyocyte cell cycle arrest. Here we describe the association of familial hypertrophic cardiomyopathy in Sphynx cats with a novel *ALMS1* mutation.

**Results:**

A G/C variant was identified in exon 12 (human exon 13) of the *ALMS1* gene in affected cats and was positively associated with the presence of hypertrophic cardiomyopathy in the feline population (*p* < 0.0001). The variant was predicted to change a highly conserved nonpolar Glycine to a positively charged Arginine. This was predicted to be a deleterious change by three in silico programs. Protein prediction programs indicated that the variant changed the protein structure in this region from a coil to a helix. Light microscopy findings included myofiber disarray with interstitial fibrosis with significantly more nuclear proliferative activity in the affected cats than controls (*p* < 0.0001).

**Conclusion:**

This study demonstrates a novel form of cardiomyopathy associated with *ALMS1* in the cat. Familial hypertrophic cardiomyopathy is a disease of genetic heterogeneity; many of the known causative genes encoding for sarcomeric proteins. Our findings suggest that variants in genes involved with cardiac development and cell regulation, like the *ALMS1* gene, may deserve further consideration for association with familial hypertrophic cardiomyopathy.

## Background

Familial hypertrophic cardiomyopathy is a very common inherited cardiovascular disorder in people, with a prevalence of 1:250 to 500 [1]. Affected individuals are at risk of developing congestive heart failure or sudden cardiac death, although many can remain stable for years [1]. A significant number of causal mutations have been identified, particularly in sarcomeric or sarcomeric associated genes, but it has been estimated that about 40% of cases are due to mutations in genes in which the association with cardiomyopathy has yet to be identified, genes referred to as the “missing causal genes” [2, 3]. Both syndromic and nonsyndromic forms of hypertrophic cardiomyopathy exist.

Mutations in the *ALMS1* gene are associated with the development of Alstrom syndrome in human beings, a multisystem familial disease that can include retinal degeneration, obesity, neurosensorial deafness, type 2 diabetes and cardiomyopathy [4–6]. Two forms of cardiomyopathy are most commonly described with Alstrom syndrome; dilated cardiomyopathy which is most frequently observed in infants; and a restrictive cardiomyopathy observed more commonly in adults [6, 7]. Cardiomyopathy associated with mutations in the *ALMS1* gene is most often observed in conjunction with other multisystemic effects of Alstrom syndrome, however, some patients develop isolated cardiomyopathy in the absence of multisystemic disease [5, 6, 8]. Hypertrophic cardiomyopathy has not yet been described.

This study describes the discovery of a mutation in the *ALMS1* gene associated with the development of familial hypertrophic cardiomyopathy in the domestic cat, an excellent model of human hypertrophic cardiomyopathy due to many similarities, including the familial nature, clinical presentation and pathologic findings [9, 10].

## Results

After filtering for variants that were present in at least 50% of the affected Sphynx and in 5% or less of the unaffected control cats, two hundred and forty-seven variants in 189 genes were predicted to be of moderate or high impact (frameshift, nonsynonymous variant, codon insertion, codon deletion, stop gained or lost, start gained or lost, splice site acceptor or donor). None of these variants were identified in the common hypertrophic cardiomyopathy causative genes including β myosin heavy chain, myosin binding protein 3, cardiac troponin I, myosin regulatory light chain, myosin essential light chain, α-tropomyosin, cardiac α actin [11].

One hundred and seventy-four variants believed to have a likely cardiac impact or influence were evaluated by Sanger sequencing (Additional file 1: Supplemental Data Table 1) and excluded by presence in more than 5% of the control population alleles or presence in less than 50% of the affected Sphynx cat population.

**Fig. 1:**
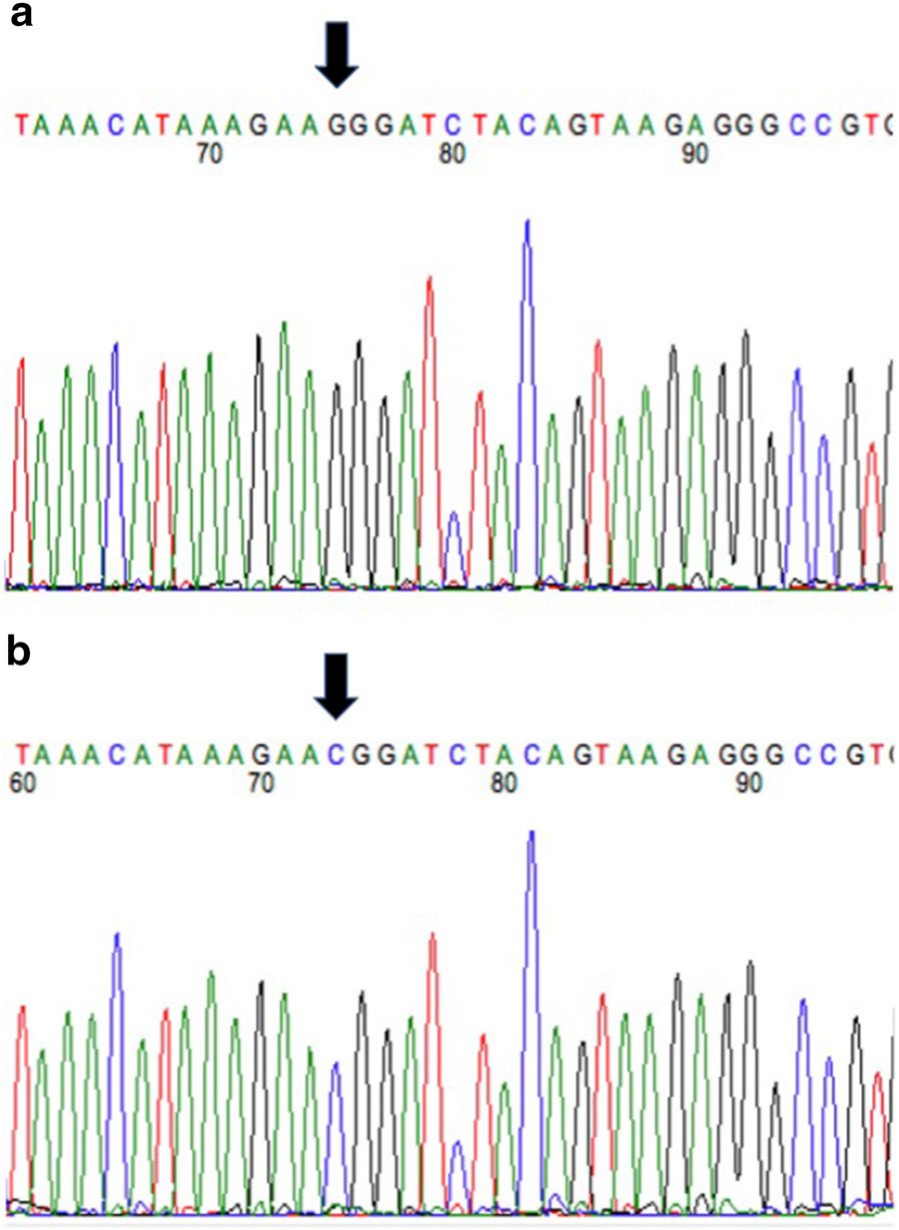
**a** Chromatogram of DNA sequence from unaffected cat. Arrow indicates location of DNA variant (G in unaffected cat). **b** Chromatogram of DNA sequence from affected cat homozygous for the *ALMS1* variant. Arrow indicates location of DNA variant (C in affected cat)

**Fig. 2:**
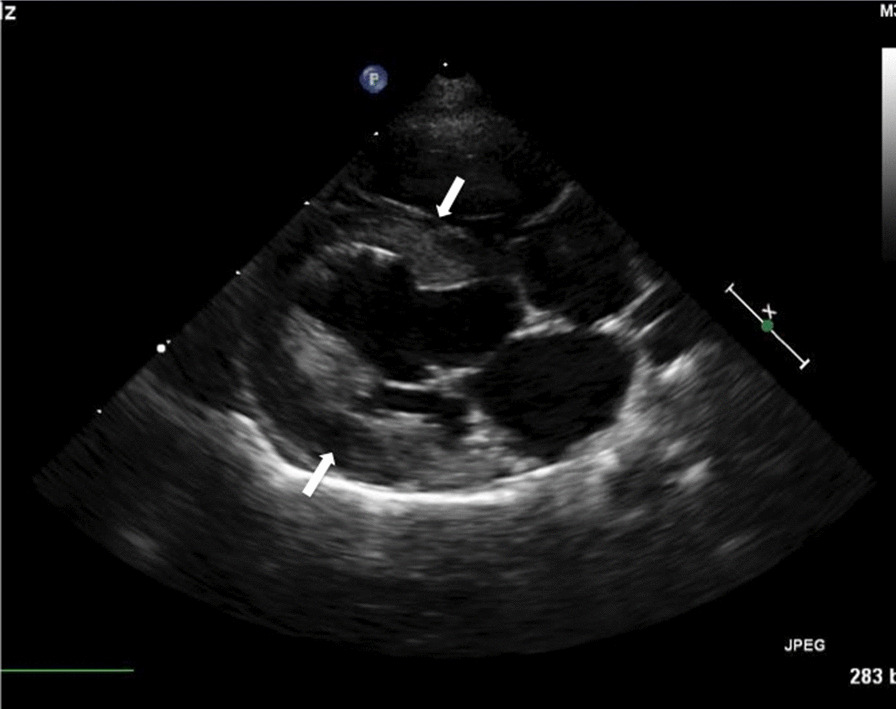
Long axis 4 chamber view of an affected Sphynx cat demonstrating left ventricular free wall and interventricular septal hypertrophy denoted by arrows. The scale marker is equivalent to 1 cm

A G/C variant was observed in the *ALMS1* gene in affected Sphynx cats at A3: 92439157 (ENSFCAG00000008756) in feline exon 12 (human orthologue of exon 13). (Fig. 1) The variant is predicted to change the amino acid from a highly conserved Glycine, a nonpolar amino acid, to a positively charged Arginine (Additional file 2: Supplemental Data Table 2). (Fig. 2) The variant was positively associated with the presence of feline hypertrophic cardiomyopathy in the cat population with a *p* value of < 0.0001 and was identified in 62 (27 heterozygotes, 35 homozygotes) of 71 affected Sphynx. Affected cats ranged from 1 to 14 years of age (mean of 5 years) and included 19 females and 42 males. The other nine (1–6 years of age; 4 females, 5 males) affected Sphynx cats did not have either the ALMS1 variants or the two known feline hypertrophic cardiomyopathy *MYBPC3* mutations [21, 22]. The variant was only found in 2 (both heterozygotes) of 214 non-sphynx cats without known heart disease for an allele frequency of 0.4% in this general cat population. The variant had a penetrance of 77% with a relative risk of 13.6 within the feline population.

**Fig. 3:**
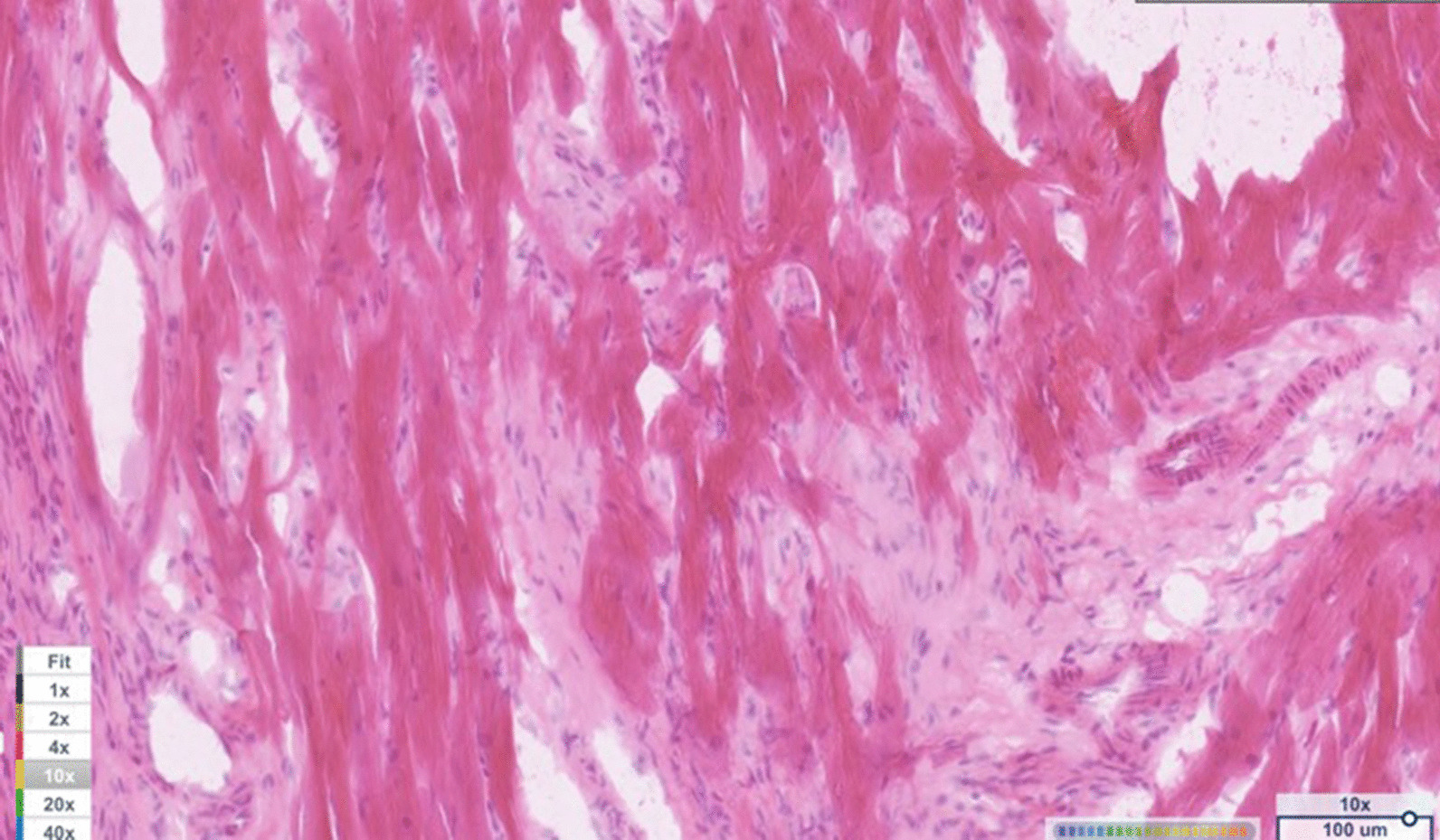
Light microscopy findings included myofiber disarray with interstitial fibrosis. H & E staining at 10 × magnification

The variant was predicted to be a deleterious change by all three mutation prediction algorithms. Polyphen (http://genetics.bwh.harvard.edu/pph2/), predicted the mutation to be likely damaging (score of 1.0) (Fig. 3); SIFT (http://sift.jcvi.org/) predicted it to be a deleterious change (score of 0.0) and Provean (http://provean.jcvi.org/index.php) predicted it to be a deleterious change (score of − 6.923).

Protein prediction programs indicated that the variant changed the protein structure from an area of a coil to an area of a helix.

**Fig. 4:**
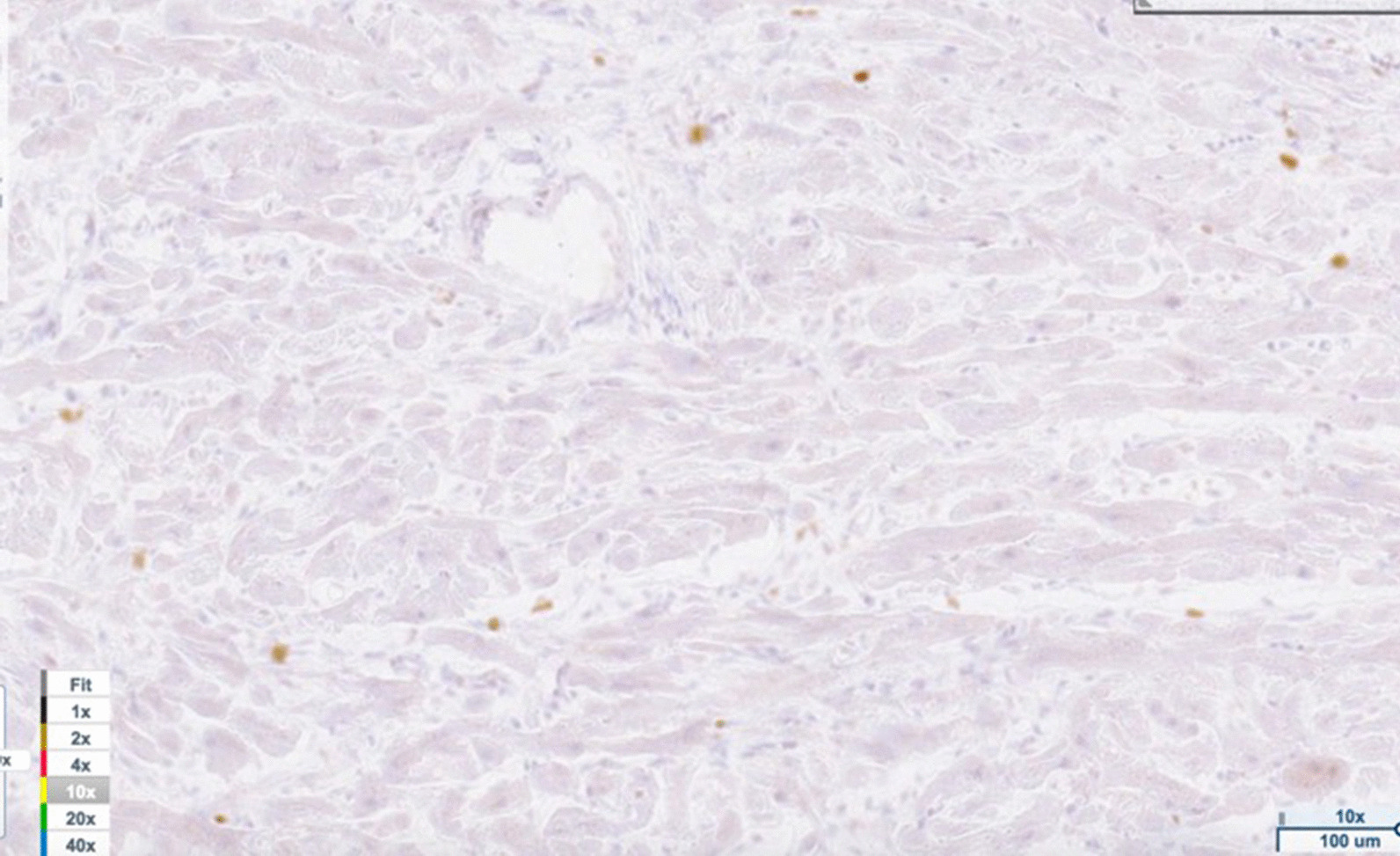
Ki67 staining (brown) of nuclear proliferative activity in myocardium of affected Sphynx cat at 10 × magnification

**Fig. 5:**
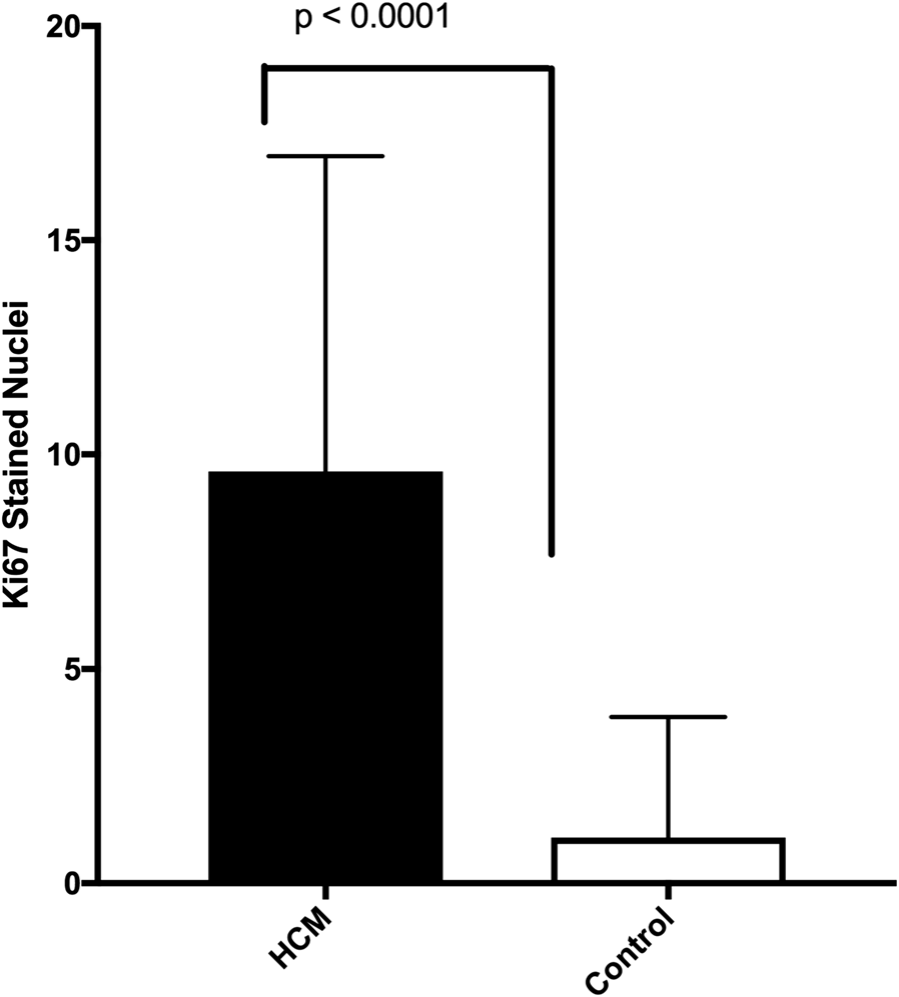
Ki67 staining for nuclear proliferative activity was significantly greater in the affected cats than controls (mean of 10 stained nuclei per cat in affected cats, mean of 1 stained nuclei per cat for control cats) (*p* < 0.0001)

Light microscopy findings included myofiber disarray with interstitial fibrosis. (Fig. 3) Ki67 staining for nuclear proliferative activity of each cat was significantly greater in the affected cats than controls (mean of 10 stained nuclei for HCM, mean of 1 for controls). (*p* < 0.0001). (Figs. 4, 5) There was no co-labeling with vimentin and Ki67.

## Discussion

This is the first report of a variant in the Alstrom syndrome protein 1 (*ALMS1)* gene in a domestic animal and the first report of an *ALMS1* variant associated with the development of hypertrophic cardiomyopathy. The *ALMS1* gene is a large gene that encodes for a ubiquitously expressed protein [12]. The function of the protein is not well understood although it is believed to be associated with energy metabolism and homeostasis, intracellular trafficking, cell signaling pathways, cell differentiation and cell cycle control [5]. Of particular interest may be the role of the ALMS1 protein in the regulation of cell cycle proliferation in perinatal cardiomyocytes [13]. Although cardiomyocyte cell division and replication in mammals generally declines soon after birth, inhibition of ALMS1 expression in mice lead to increased cardiomyocyte proliferation, and deficiency of Alstrom protein has been suggested to impair post-natal cardiomyocyte cell cycle arrest. Additionally, a mitogenic cardiomyopathy characterized by an increase in number of cardiomyocytes in mitosis was observed in two infants with Alstrom syndrome [14]. Since the Ki67 antibody has been used to assess cardiomyocyte nuclear proliferative activity in mitogenic cardiomyopathy, it was used to assess the nuclear activity in the Sphynx cats with the *ALMS1* variants and hypertrophic cardiomyopathy reported here [15]. We demonstrated that cats with hypertrophic cardiomyopathy and the variant in the *ALMS1* gene had significantly more myocyte nuclear activity than the control cats. This suggests that hypertrophic cardiomyopathy in this feline model may be associated with impaired myocyte cell cycle arrest.

The forms of cardiomyopathy typically identified in individuals with ALMS syndrome include dilated or restrictive cardiomyopathy [7]. The cats reported here had hypertrophic cardiomyopathy. There are other examples where mutations within the same gene can lead to the development of hypertrophic, dilated or restrictive cardiomyopathy [16, 17]. This pleiotropic effect may be associated with location of the variant within the specific gene and the effect of variant location on interactions with other proteins at this region [3]. In fact, variants in the *ALMS1* gene associated with the development of dilated cardiomyopathy have been more commonly reported in exons 8, 10 and 16, while the hypertrophic cardiomyopathy variant identified here was in exon 13 [18]. It is possible that variants in this exon could impact the protein in a way that is more consistent with the development of hypertrophic cardiomyopathy.

In people, mutations in the *ALMS1* gene are most frequently associated with multisystem disease, including retinal degeneration, obesity, neurosensorial deafness, type 2 diabetes as well as cardiomyopathy [4, 5]. In the cats reported here, none of the multisystem issues appeared to have been observed, although mild forms of these issues including deafness, obesity and even retinal degeneration may have been less apparent in a domestic cat living in a well-controlled environment. However, isolated cardiac disease, as apparently observed in this feline model, has also been noted [5, 6, 8].

Not all Sphynx cats with hypertrophic cardiomyopathy evaluated here had the *ALMS1* variants. The Sphynx cat is a purebred hairless cat developed from a genetic mutation responsible for the hairless phenotype. To allow increased heterozygosity in the breed, the cat is intentionally outbred to other domestic cats every few generations. It is possible that the affected Sphynx cats without the *ALMS1* variant obtained a different causative variant from the general cat population since it has been estimated that approximately 15% of domestic cats suffer from hypertrophic cardiomyopathy [19]. Two genetic mutations have been previously reported in the domestic cat but neither was present in the remaining cats, suggesting there is likely at least one additional mutation in the cat [20, 21].

A limitation of this study is that we compared and validated the *ALMS1* variant in the affected Sphynx cat population to a population of unaffected non-Sphynx cats rather than a population of unaffected Sphynx cats. Hypertrophic cardiomyopathy is a disease of variable penetrance and expression both in human beings and cats [9, 11]. The affected Sphynx cats studied here were diagnosed between the ages of 1 and 14 years of age. The highly variable age of onset and severity of disease complicates the phenotyping and identification of truly unaffected Sphynx cats and contributed to the decision to compare sequence data to a population of unaffected cats of several different cat breeds rather than unaffected Sphynx cats. The failure to evaluate a population of unaffected Sphynx cat limits the ability to state with certainty that the *ALMS1* variant is not a breed related polymorphism however the absence of this variant in 200 cats from 14 other cat breeds, the predicted deleterious impact of the variant in a highly conserved amino acid and the histopathologic findings of increased nuclear proliferative activity in the myocardium of affected cats with this variant would support the association of the *ALMS1* variant and the development of disease. Future prospective studies that follow cats with this variant over multiple years will improve our understanding of this disease.

## Conclusions

Here we describe the association of familial hypertrophic cardiomyopathy in Sphynx cats with a novel *ALMS1* mutation. Familial hypertrophic cardiomyopathy is a disease of genetic heterogeneity with many of the known causative genes encoding for proteins that have a role in sarcomeric function. However, approximately 40% of human cases have a disease of an unknown causal variant [2]. Our findings suggest that variants in genes involved with other stages of cardiac development and cell regulation, like the *ALMS1* gene, may deserve further consideration for association with familial hypertrophic cardiomyopathy. The findings here demonstrate an additional gene associated with the feline disease, however the absence of this mutation in some of the affected cats indicate at least one additional mutation is yet to be identified.

## Methods

Eighty-one Sphynx cats diagnosed with hypertrophic cardiomyopathy by an echocardiographic examination performed by a board-certified veterinary cardiologist were recruited for evaluation. Cats were considered to be affected if they had a left ventricular end diastolic wall thickness of at least 0.6 cm for the interventricular septum and/or left ventricular free wall [22]. Thirteen domestic short haired cats over the age of 10 years were selected to serve as controls after evaluation by a board-certified veterinary cardiologist and being cleared of cardiomyopathy based on an echocardiographic identification of a left ventricular end diastolic wall dimension of less than 0.4 cm for the interventricular and/or left ventricular free wall.

DNA samples were obtained from each affected Sphynx cat and control cat. DNA samples from 214 cats with no known history of cardiac disease from 14 breeds (Bengal, Birman, British short hair, Burmese, Domestic Short Hair, Domestic Long Hair, Himalayan, Maine Coon, Manx, Norwegian Forest Cat, Persian, Ragdoll, Scottish Fold, Siamese) maintained in an archive at the NCSU College of Veterinary Medicine were also available for analysis. Approximately 3 µg of DNA from 14 affected Sphynx cats and the 13 control cats was submitted for library preparation and whole genome sequencing (WGS) at the University of North Carolina Chapel Hill High-Throughout Sequencing Facility (https://www.med.unc.edu/genomics). All sequencing experiments were designed as 125 base paired-end reads and samples were run on either 1 or 2 lanes of an Illumina HiSeq 2500 high-throughput sequencing system.

Read alignment and variant calling from WGS data were performed using a standardized bioinformatics pipeline for all samples as follows. Raw sequencing reads were quality checked for potential sequencing issues and contaminants using FastQC (http://www.bioinformatics.babraham.ac.uk/projects/fastqc/). Adapter sequences, primers, Ns, and reads with quality score below 28 were trimmed using Trimmomatic [23, 24]. Reads with a remaining length of fewer than 20 base pairs after trimming were discarded. Post-trimmed reads were mapped to the *Felis catus* (Felis_catus 6.2) reference genome using BWA-MEM [25, 26]. Duplicated reads were marked using SAMBLASTER, and variants were called with Platypus [27–29]. The detected variants were annotated with dbSNP ID when available in dbSNP for *Felis catus* release 127 and variant effects were predicted with SnpEff based on Ensembl gene model release 75.

Variants present in affected cats were selected and filtered against a database of variants from the 13 control cats using the Variant Explorer tool of the Maverix Analytic Platform (http://www.maverixbio.com/). Variants were then categorized by Variant Effect Predictor 87 and prioritized for further study by their functional impact (e.g., stop codon, frameshift, indel, etc.) as well as potential cardiac involvement based on presence in a gene previously associated with a form of cardiomyopathy or a gene known to encode for proteins expressed in the myocardium, particularly those thought to be associated with myocardial function or development (https://www.qiagenbioinformatics.com/products/ingenuity-pathway-analysis/).

Variants were then evaluated for likely pathogenic implications using the Standards and Guidelines for the Interpretation of Sequence Variants [30]. First, variants observed in more than 5% of the control population alleles were excluded. Next, a sequence was considered pathogenic if it was likely to be a truncating variant including gain of a stop codon, a frameshift indel, an altered splice site, an altered start codon, and/or a single or multi-exon deletion. Missense variants were considered to be significant if they altered a well conserved amino acid and were judged to have a likely deleterious impact with at least 2 of 3 in-silico programs including Polyphen (http://genetics.bwh.harvard.edu/pph2/), Sift (http://sift.jcvi.org/) and Provean (http://provean.jcvi.org/index.php). Finally, the data was visually inspected for multiallelic variations including larger insertions and deletions using the GenomeBrowse 2.1.2 (http://goldenhelix.com/products/GenomeBrowse/index.html) visualization tool.

The most promising variants were selected for further evaluation by Sanger Sequencing of DNA from 68 affected Sphynx diagnosed as previously described and the 214 cats with no known history of cardiac disease (controls). Finally, the variants were tested for allelic association with hypertrophic cardiomyopathy using a Fisher’s exact test. A *p* value of < 0.05 was considered significant. Penetrance and relative risk were determined for each of the most promising variants that were evaluated by Sanger Sequencing.

To better determine the potential impact of the variant at the muscular level, immunohistochemistry was performed for Ki67, a known marker of nuclear proliferative activity, and vimentin, to determine if any nuclear proliferative activity was associated with fibroblast or myocyte cells. More specifically, myocardial samples were collected immediately at time of death from 4 affected Sphynx cats and 3 mature non-sphynx control cats euthanized for non-cardiac disease. Samples were snap frozen at − 80° and 10 um cryostat sections were placed on charged glass slides. After fixing with 10% neutral buffered formalin, heat-induced epitope retrieval was performed (Thermo, #TA-135-HBL), and endogenous peroxidase activity was quenched with 3% hydrogen peroxide. Tissues were blocked in 10% normal goat serum for one hour, then incubated overnight at 4 °C with rabbit anti-Ki67 antibody (Vector Laboratories, #VP-RM04; 1:100), followed by incubation with biotinylated goat anti-rabbit secondary antibody (Jackson #111-065-144; 1:500) for 1 h at room temperature. Signal was amplified using a Vectastain Elite ABC-HRP kit (Vector Laboratories, #PK-6100), and visualized using DAB (Thermp, #TA-QHDX-125). Tissues were blocked again in 10% normal goat serum for one hour, then incubated overnight at 4 °C with mouse anti-Vimentin antibody (Sigma, #V5255; 1:100), followed by incubation with biotinylated goat anti-mouse secondary antibody (Jackson #115-065-166) for one hour at room temperature. Signal was amplified using a Vectastain Elite ABC-AP kit (Vector Laboratories, #AK-5000), and visualized using Immpact Vector Red (Vector Laboratories, #SK-5105). Finally, tissues were counterstained with Hematoxylin, dehydrated using a series of graded alcohols, cleared with xylene and coverslipped with DPX (Electron Micrscopy Sciences, #13512). Using light microscopy, the amount of proliferative activity was evaluated blindly by one investigator (KM) by photographing each slide 5 times, randomizing the images and counting the number of Ki67 stained nuclei in 5 predefined areas (4 borders, center) of each slide at 4 × magnification. The number of stained nuclei was summed for each slide. The number of stained nuclei was compared between control cats and hypertrophic cardiomyopathy cats using the Student’s t test, with *p* < 0.05 considered to be significant.

## Supplementary Information

**Additional file 1: Supplemental Table 1.** Variants pursued by Sanger Sequencing.

**Additional file 2: Supplemental Table 2.** Amino Acid Conservation.

## Data Availability

The datasets used and analysed during the current study are available from the corresponding author on reasonable request.
