# Supplementary material for: A deleterious mutation in the ALMS1 gene in a naturally occurring model of hypertrophic cardiomyopathy in the Sphynx cat
Source: Orphanet J Rare Dis. 2021 Feb 27;16:108. doi: 10.1186/s13023-021-01740-5 (PMC7913409; doi:10.1186/s13023-021-01740-5)
Supplement: Supplementary file 2 — Additional file 2: Supplemental Table 2. Amino Acid Conservation. [file 13023_2021_1740_MOESM2_ESM.docx]

Felis Catus (Cat) **ALMS1 Variant** PA D T K H K E R I Y S K R

Felis Catus (Cat) PA D T K H K E G I Y S K R

Canis Familiaris (Dog) P A N I K H K E G I Y S K R

Bos Taurus (Cow) T V N I K H K E G I Y S K R

Homo Sapien (Human) T V N I K H K E G I Y S K R

Mus musculus (Mouse) T V N I K H K E G I Y S K R

Ursus americanus (Bear) T D N M K H K E G I Y S K R

Sus scrofa (Pig) A V N I K H K E G I Y S K R

[Loxodonta africana (African elephant)](https://www.uniprot.org/taxonomy/9785) D V S I K H K E G I Y S K R

Rhinolophus ferrumequinum (Bat) T V N I K H K E G I Y S K R
